# Activity In Vitro of Clotrimazole against Canine Methicillin-Resistant and Susceptible *Staphylococcus pseudintermedius*

**DOI:** 10.3390/antibiotics6040029

**Published:** 2017-11-22

**Authors:** Sian-Marie Frosini, Ross Bond

**Affiliations:** Clinical Science and Services, Royal Veterinary College, Hawkshead Lane, North Mymms, Hatfield AL9 7TA, UK; rbond@rvc.ac.uk

**Keywords:** *Staphylococcus pseudintermedius*, clotrimazole, canine, otitis externa, topical therapy

## Abstract

Emergence of multidrug-resistance in *Staphylococcus pseudintermedius* (SP) has increased interest in topical therapy as an alternative to systemic antibiotics in canine pyoderma. The antifungal imidazole, clotrimazole, is contained in numerous licensed canine ear preparations. Its in vitro activity against SP has not been evaluated, although previous studies have shown that the related imidazole, miconazole, has significant anti-staphylococcal efficacy. We therefore determined minimum inhibitory concentrations (MICs) of clotrimazole amongst 50 SP isolates (25 methicillin-resistant [MR]SP and susceptible [MS]SP) collected from dogs in Germany during 2010–2011 using an agar dilution method (CLSI VET01-A4). MICs amongst MRSP and MSSP were comparable (MIC_50_ and MIC_90_ = 1mg/L for both groups, *p* = 0.317); overall, 49 isolates had MIC = 1 mg/L and one had MIC = 0.5 mg/L. The relatively low MICs obtained in this study are likely to be exceeded by topical therapy and thus further clinical evaluation of clotrimazole use in canine superficial pyoderma and otitis externa caused by MRSP and MSSP is now warranted.

## 1. Introduction

*Staphylococcus pseudintermedius* is routinely isolated from canine otitis externa and superficial pyoderma, two of the most common dermatological diseases encountered by first opinion veterinary practitioners [1,2]. The emergence of methicillin-resistant *S. pseudintermedius* (MRSP), resistant to most or all systemically licensed antimicrobials available to veterinary practitioners [3], paralleled by recognition of the risk of zoonotic infections for pet owners [4,5], has led to increased interest in a topical therapeutic approach using narrow-spectrum antimicrobials [6].

Clotrimazole is a broad-spectrum anti-fungal imidazole that is widely used in human medicine for the topical therapy of dermatophytosis, vulvovaginal and oropharyngeal candidiasis [7]. It is also a component of polypharmaceutical ear drops licensed for use in dogs in various European countries; this is primarily due to its activity against *Malassezia pachydermatis* [8], a commensal yeast and important ear and skin pathogen in this species [9]. Clotrimazole and related imidazoles have also been shown to have a narrow spectrum of antibacterial activity, encompassing human-derived *S. aureus*, *S. epidermidis*, *Mycobacterium smegmatis* and *Streptomyces* spp., but not *Escherichia coli* and *Streptococcus pyogenes* [10,11,12,13]. Clotrimazole and miconazole had identical and low minimum inhibitory concentrations (MICs) (1 mg/L) when tested against a single reference strain of *S. aureus* [12] and methicillin-resistant *S. aureus* isolates from human burn patients were also susceptible to clotrimazole [14]. Previous studies have shown good in vitro efficacy of miconazole against canine-derived *S. pseudintermedius*, including MRSP [15,16,17], whereas the in vitro activity of clotrimazole against canine-derived *S. pseudintermedius* does not appear to have been reported. We therefore determined the in vitro susceptibility of canine-derived European MRSP isolates and their methicillin-susceptible counterparts (MSSP) to clotrimazole using an agar dilution method. 

## 2. Materials and Methods 

Coagulase-positive staphylococci (25 MRSP and 25 MSSP obtained in 2010–2011) isolated from clinical infections of dogs were randomly selected from our collection of staphylococci previously used for a canine risk factor study [18]. Species identification and methicillin resistance were previously confirmed using both phenotypic and genotypic (*nuc*, *mecA*) methods [16]. 

MICs were determined in duplicate by agar dilution (CLSI VET01-A4) [19]. Before MIC determination, strains were subcultured twice on blood agar base (CM0271, Oxoid, Basingstoke, UK) containing 5% sheep blood (TCS Biosciences, Buckingham, UK) at 35 °C for 24 h. Stock concentrations of 10× final concentration of clotrimazole (PHR1058, Sigma-Aldrich, Gillingham, UK) were prepared in DMSO (Sigma-Aldrich), adjusted for drug potency [19]. Final concentration of active fraction of clotrimazole in agar plates ranged from 0.125 to 32 mg/L in two-fold dilutions. Discrepancy between duplicate MICs was accepted, provided they varied by only one dilution; the higher value was identified as the MIC in these cases. For quality control purposes, *S. pseudintermedius* LMG 22219 and *S. intermedius* ATCC 29663 were included. Three UK staphylococcal isolates with previous variable miconazole MICs ranging from 0.5–256 mg/L were also included for comparative purposes [16,17].

Normality of data distribution was assessed using the Shapiro-Wilk test. MIC values for MRSP and MSSP were compared using the Mann-Whitney U test incorporating Holm-Bonferroni adjustments using the SPSS version 24 statistical software package (IBM UK Ltd, Portsmouth, UK), with *p* < 0.05 for significance.

## 3. Results

The MICs of clotrimazole were remarkably uniform, with 49/50 isolates having MICs of 1 mg/L (*n* = 1 MSSP, MIC = 0.5 mg/L) (Table 1). There was no significant difference (*p* = 0.317) between MICs for MRSP and MSSP (for both groups MIC_50_ = 1 mg/L, MIC_90_ = 1 mg/L). MICs for LMG 22219 and ATCC 29663 were 1 mg/L and thus closely comparable to those of the wider collection of field isolates (Table 2). MICs of clotrimazole for the isolates with variable MICs of miconazole were 2, 1 and 2 mg/L, respectively (Table 2). On no occasions did the MICs vary between replicates. Appropriate growth was observed for all isolates in both start and end control plates.

## 4. Discussion

Topical skin pharmaceuticals with broad activity against staphylococci and *Malassezia* yeasts are of particular interest in canine dermatology in view of the frequent co-existence of these pathogens in infected skin and ears [9]. These infections commonly relapse due to the difficulties in correcting predisposing factors such as underlying hypersensitivity diseases (especially canine atopic dermatitis) and anatomical features, such as skin folds or hairy or occluded external ear canals. Development of well-tolerated topical antibiotic and antiseptic preparations, that effectively control these relapsing infections without promoting resistance, is an important objective in the context of responsible antimicrobial use, wherein limiting the use of systemic therapy is key [12]. Our study indicates that clotrimazole is a pharmaceutical agent that warrants further consideration in veterinary dermatology as a topical anti-staphylococcal molecule, in addition to its well-recognised antifungal properties.

The uniformly low MICs of clotrimazole throughout our sizeable collection of canine-derived *S. pseudintermedius* isolates were comparable to those reported previously for the closely-related imidazole, miconazole [15,16,17]. MIC values were also similar to those reported for clotrimazole, miconazole and econazole against a single reference strain of human-derived *S. aureus* (all 1 mg/L) [12], and for 18 strains of MRSA obtained from human burn patients in China (MIC_50_ and MIC_90_ = 2 mg/L) [14]. The comparable efficacy against both MSSP and MRSP in this study indicates that methicillin-resistance does not influence susceptibility to clotrimazole in vitro; clinical efficacy should therefore be expected in both canine and human skin infections with methicillin-resistant staphylococci. This hypothesis should be now tested in clinical studies. 

In existing European-licensed topical ear formulations for dogs, clotrimazole is combined with a glucocorticoid (dexamethasone or betamethasone) and either marbofloxacin (a veterinary fluoroquinolone) or gentamicin. The role of conventional susceptibility testing of bacterial pathogens for the canine ear canal is somewhat contentious since very high concentrations of antibiotic drug can be achieved locally using topical therapy, leading to clinical efficacy in cases where “resistance”, determined by breakpoints based on tissue concentrations following systemic use, is reported by the diagnostic laboratory. Whilst considering this limitation, approximately 88% of 103 isolates from a global collection of MRSP carried resistance genes for both fluoroquinolones and gentamicin [20]. The presence of clotrimazole (or miconazole) in an ear product might therefore provide anti-bacterial and clinical efficacy in cases of MRSP infection when the combined antibiotic would otherwise be ineffective. Similar efficacy might be expected should clotrimazole-containing products be developed and evaluated for use in wider skin infections caused by staphylococci and *Malassezia* yeasts, as a desirable alternative to oral antibiotic treatment in dogs.

## 5. Conclusions

The relatively low MICs of clotrimazole obtained in this study are likely to be exceeded by topical therapy and thus further clinical evaluation of clotrimazole use in canine superficial pyoderma and otitis externa caused by MRSP and MSSP is now warranted.

## Figures and Tables

**Table 1:** Minimum inhibitory concentrations (MICs) of clotrimazole for 50 *Staphylococcus pseudintermedius* isolates (25 methicillin-resistant *S. pseudintermedius* (MRSP), 25 methicillin-susceptible *S. pseudintermedius* (MSSP)) from dogs established using an agar dilution method.

Bacterial Type	MIC (mg/L)									MIC_50_ (mg/L)	MIC_90_ (mg/L)
	0.125	0.25	0.5	1	2	4	8	16	32		
MRSP	0	0	0	25	0	0	0	0	0	1	1
MSSP	0	0	1	24	0	0	0	0	0	1	1
Total	0	0	1	49	0	0	0	0	0	1	1

MRSP: methicillin-resistant *S. pseudintermedius;* MSSP: methicillin-susceptible *S. pseudintermedius*.

**Table 2:** MICs (mg/L) of clotrimazole and miconazole for strains/isolates of *Staphylococcus aureus*, *S. intermedius* and *S. pseudintermedius* used for quality control and comparative purposes.

Bacterial Type	Designation	MIC (mg/L)		
		Miconazole [15]	Miconazole [16]	Clotrimazole
*S. pseudintermedius*	LMG 22219	1	0.5	1
*S. intermedius*	ATCC 25923	1	1	1
MRSA	A057	2	ND	2
MSSP	DT050	16	0.5	1
MSSA	B122	256	1	2

MRSA, methicillin-resistant *S. aureus*; MSSA, methicillin-susceptible *S. aureus*; MSSP, methicillin-susceptible *S. pseudintermedius;* ND, not done.
